# MicroRNA and mRNA profiling in the idiopathic inflammatory myopathies

**DOI:** 10.1186/s41927-020-00125-8

**Published:** 2020-06-10

**Authors:** Joanna E. Parkes, Anastasia Thoma, Adam P. Lightfoot, Philip J. Day, Hector Chinoy, Janine A. Lamb

**Affiliations:** 1 Centre for Epidemiology, Division of Population Health, Health Services Research & Primary Care, Faculty of Biology, Medicine and Health, Manchester Academic Health Science Centre, University of Manchester, Manchester, UK; 2 Stopford Building, University of Manchester, Oxford Road M13 9PT, Manchester, UK; 3 Musculoskeletal Science & Sports Medicine Research Centre, School of Healthcare Science, Manchester Metropolitan University, Manchester, UK; 4 Manchester Institute of Biotechnology, University of Manchester, Manchester, UK; 5 Division of Evolution & Genomic Sciences, University of Manchester, Manchester, UK; 6 Centre for Musculoskeletal Research, Division of Musculoskeletal & Dermatological Sciences, Faculty of Biology, Medicine and Health, Manchester Academic Health Science Centre, University of Manchester, Manchester, UK; 7 National Institute for Health Research Manchester Biomedical Research Centre, Manchester University NHS Foundation Trust, Manchester Academic Health Science Centre, University of Manchester, Manchester, UK; 8 Department of Rheumatology, Salford Royal NHS Foundation Trust, Manchester Academic Health Science Centre, Salford, UK

**Keywords:** Idiopathic inflammatory myopathies, Polymyositis, Dermatomyositis, microRNA, RNA sequencing

## Abstract

**Background:**

The idiopathic inflammatory myopathies (IIMs) are heterogeneous autoimmune conditions of skeletal muscle inflammation and weakness. MicroRNAs (miRNAs) are short, non-coding RNA which regulate gene expression of target mRNAs. The aim of this study was to profile miRNA and mRNA in IIM and identify miRNA-mRNA relationships which may be relevant to disease.

**Methods:**

mRNA and miRNA in whole blood samples from 7 polymyositis (PM), 7 dermatomyositis (DM), 5 inclusion body myositis and 5 non-myositis controls was profiled using next generation RNA sequencing. Gene ontology and pathway analyses were performed using GOseq and Ingenuity Pathway Analysis. Dysregulation of miRNAs and opposite dysregulation of predicted target mRNAs in IIM subgroups was validated using RTqPCR and investigated by transfecting human skeletal muscle cells with miRNA mimic.

**Results:**

Analysis of differentially expressed genes showed that interferon signalling, and anti-viral response pathways were upregulated in PM and DM compared to controls. An anti-Jo1 autoantibody positive subset of PM and DM (*n* = 5) had more significant upregulation and predicted activation of interferon signalling and highlighted T-helper (Th1 and Th2) cell pathways. In miRNA profiling miR-96-5p was significantly upregulated in PM, DM and the anti-Jo1 positive subset. RTqPCR replicated miR-96-5p upregulation and predicted mRNA target (*ADK, CD28* and *SLC4A10*) downregulation. Transfection of a human skeletal muscle cell line with miR-96-5p mimic resulted in significant downregulation of *ADK*.

**Conclusion:**

MiRNA and mRNA profiling identified dysregulation of interferon signalling, anti-viral response and T-helper cell pathways, and indicates a possible role for miR-96-5p regulation of *ADK* in pathogenesis of IIM.

## Background

The idiopathic inflammatory myopathies (IIMs) are a heterogeneous group of autoimmune conditions characterised by weakness and inflammation of skeletal muscle. Major clinical classifications of IIM include polymyositis (PM), dermatomyositis (DM) and inclusion body myositis (IBM). Between 60 and 70% of IIM patients have detectable myositis associated or specific autoantibodies which are associated with particular clinical phenotypes [1].

MicroRNAs (miRNAs) are short, non-coding, single-stranded RNAs which target particular mRNAs for translational suppression and/or degradation. In this manner miRNAs ‘fine-tune’ gene expression and influence a wide variety of cellular processes and pathways. MiRNAs which regulate the immune system have been found to be dysregulated in autoimmune conditions including IIM [2] and miRNAs important in the development and maintenance of skeletal muscles, termed myomiRs, have been found to be dysregulated in IIM [3, 4]. MiRNA dysregulation has been investigated in IIM subtypes in a variety of tissues but further research is required to understand the role of microRNA in IIM pathology [5].

In this study we used next generation RNA sequencing to generate a comprehensive miRNA and mRNA profile in whole blood of PM, DM and IBM patients and then followed up particular miRNA-mRNA interactions by transfecting a human skeletal muscle cell line with miRNA mimic.

## Methods

### Study population

Patients with definite IIM were recruited as part of the Salford Royal NHS Foundation Trust myositis research tissue bank (MRTB). All patients fulfilled Bohan and Peter criteria for PM or DM [6, 7] and Griggs or European Neuromuscular Centre or Medical Research Council criteria for IBM [8, 9]. Control samples were age and ethnicity matched from MRTB taken for diagnostic or treatment purposes (non-myositis controls). All clinical data was held on the EuroMyositis database [10]. Demographic features for the 7 PM, 7 DM, 5 IBM patients and 5 non-myositis controls included in this study are summarised in Table 1. Disease duration ranged from < 1 year to 10 years at time of blood sample. On average, disease duration was slightly higher in the IBM group (4.2 years) compared to PM, DM or anti-Jo1 positive groups (< 3.2, < 1.7 and < 3.4 years respectively). One antibody negative PM patient had been diagnosed with cancer at the time of blood draw (in situ intraepithelial non-infiltrating carcinoma).

**Table 1:** Demographic and autoantibody status information for samples

	PM (*n* = 7)	DM (*n* = 7)	IBM (*n* = 5)	Control (*n* = 5)
% Female	57.1	57.1	40	20
Mean age	59.3 ± 7.2	50.6 ± 14.4	65.2 ± 4.6	44.6 ± 10.7
Myositis Specific or Associated Autoantibodies	4 anti-Jo1	1 anti-Jo1 1 anti-MDA5 1 anti-SAE 1 anti-PmScl	Negative	Negative

*PM* Polymyositis, *DM* Dermatomyositis, *IBM* Inclusion body myositis

### RNA preparation and sequencing

Total RNA was extracted from whole blood using MagMAX™ for stabilised blood tubes RNA Isolation Kit (Ambion). RNA concentration and integrity was measured using Agilent RNA 6000 Nano chips on the Bioanalyzer 2100. Complementary DNA (cDNA) library preparation was performed using NEBNext® Multiplex Small RNA Library Prep sets for Illumina using 6% PolyAcrylamide gel to perform size selection for miRNA cDNA libraries. Illumina TruSeq® Stranded mRNA library preparation kit was used for mRNA. Paired end sequencing was performed for both libraries on an Illumina HiSeq 4000. Samples were run 8 to a lane with a read depth of approximately 39 million per sample.

### RNA sequencing analysis

FastQC was used to produce quality reports for each sample (read 1 and read 2, refer to Additional file 1 for details of quality control methods). Reads were trimmed using Trimmomatic, mapped to the human genome (hg38, gencode v25) using STAR, counted using htseq, and differential gene expression was analysed using DESeq2.

### Gene ontology and pathway analyses

The R package ‘GOseq’ was used to analyse significantly differentially expressed (DE) genes and identify over or under-represented gene ontology terms (see Additional file 1 for details).

QIAGEN’s Ingenuity Pathway Analysis (IPA) (IPA®, QIAGEN Redwood City, www.qiagen.com/ingenuity) ‘Canonical Pathways’ tool was used to identify pathways with an enrichment of significantly DE genes and the ‘MicroRNA Target Filter’ tool was used to match significantly DE miRNA (*p* < 0.01) to predicted or experimentally observed mRNA targets with significant (FDR < 0.05) opposite expression changes in the mRNA RNA sequencing data (see Additional file 1 for details).

### RTqPCR

cDNA synthesis was performed using the high capacity RNA-to-cDNA™ kit for mRNA and the TaqMan® Advanced miRNA cDNA Synthesis Kit for miRNA (Applied Biosystems). RTqPCR was performed on the QuantStudio™ 12 k Flex (Applied BioSystems) using TaqMan® Gene Expression and Advanced miRNA Assays (full list of assays in Additional Table 1). Relative expression was calculated using the 2^-ΔΔCt^ method compared to reference genes (*PRDM4* and *UBE2D2* for mRNA, miR-503-5p and miR-425-5p for miRNA assays).

### Transfection of human skeletal muscle cell line

An immortalised human skeletal muscle cell line, generated from primary human myoblasts, was cultured [11] (refer to Additional file 1 for details). Cells were seeded at 40,000 cells per well and incubated at 37 °C for 24 h before transfection. Transfection was performed using Lipofectamine RNAiMAX and mirVana™ miRNA mimic for hsa-miR-96-5p (Assay ID MC10422) or negative control (Negative Control #1, Ambion). After transfection, cells were incubated at 37 °C for 24 h. Total RNA was isolated from cells using TRIzol® Reagent (Invitrogen) and gene expression measured by RTqPCR as described above.

### Statistical analyses

RNAseq and pathway analyses false discovery rates were calculated using the Benjamini-Hochberg method.

*P*-values for the RTqPCR analyses were calculated using an independent T-test on delta Ct values from each sample (Experimental Ct-Reference Ct) in the subgroups.

## Results

### Differentially expressed genes and microRNAs in IIM patients compared to controls

**Fig. 1:**
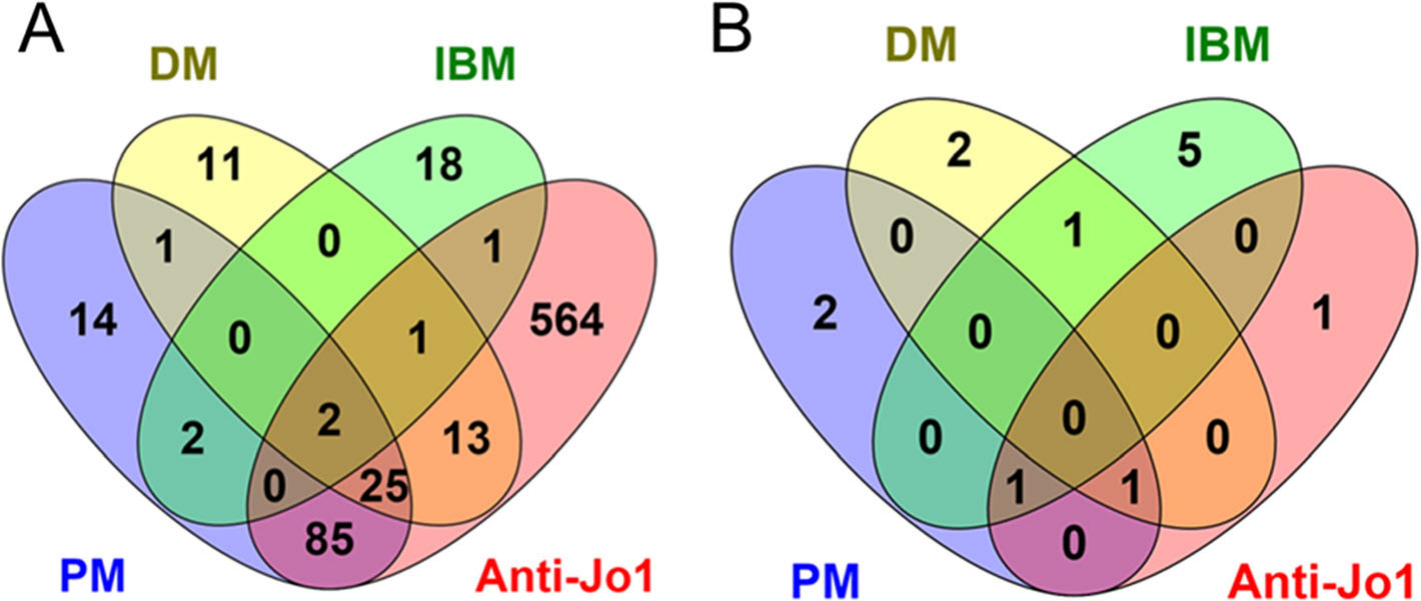
Significantly differentially expressed mRNA (**a**) and microRNA (**b**) in idiopathic inflammatory myopathy subgroups versus controls *DM* Dermatomyositis, *PM* Polymyositis, *IBM* Inclusion Body Myositis *Anti-Jo1* Anti-Jo1 autoantibody positive samples Generated from lists of significantly differentially expressed mRNA (FDR < 0.05) and microRNA (*p* < 0.01) in RNA sequencing of whole blood from 7 PM, 7 DM, 5 IBM and 5 controls (4 PM and 1 DM were anti-Jo1 autoantibody positive) using Venny 2.1 [Oliveros, J.C. (2007–2015) Venny. An interactive tool for comparing lists with Venn’s diagrams. http://bioinfogp.cnb.csic.es/tools/venny/index.html]

Overall, 129, 53 and 24 DE mRNAs were identified for PM, DM and IBM compared to controls, respectively (false discovery rate (FDR) < 0.05) (Fig. 1a, Additional file 2). Analysis of the five anti-Jo-1 positive samples (4 PM and 1 DM) compared to controls identified 691 DE genes (Fig. 1a, Additional file 2). In analysis of miRNAs, 4, 4, 7 and 3 miRNAs were DE for the PM, DM, IBM and anti-Jo-1 positive subgroups compared to controls, respectively (*p* < 0.01) (Fig. 1b, Additional file 3). No DE miRNAs were identified at FDR < 0.05.

### Innate immunity pathways are enriched for dysregulation in PM and DM and the anti-Jo1 positive subset

GOseq analysis of genes differentially expressed in the PM, DM and anti-Jo1 subgroups compared to controls found that the categories with the most significant over-representation of DE genes (Benjamini Hochberg adjusted *p*-value< 0.05) were immune-related responses including responses to type I interferon and viruses (Additional file 4).

**Fig. 2:**
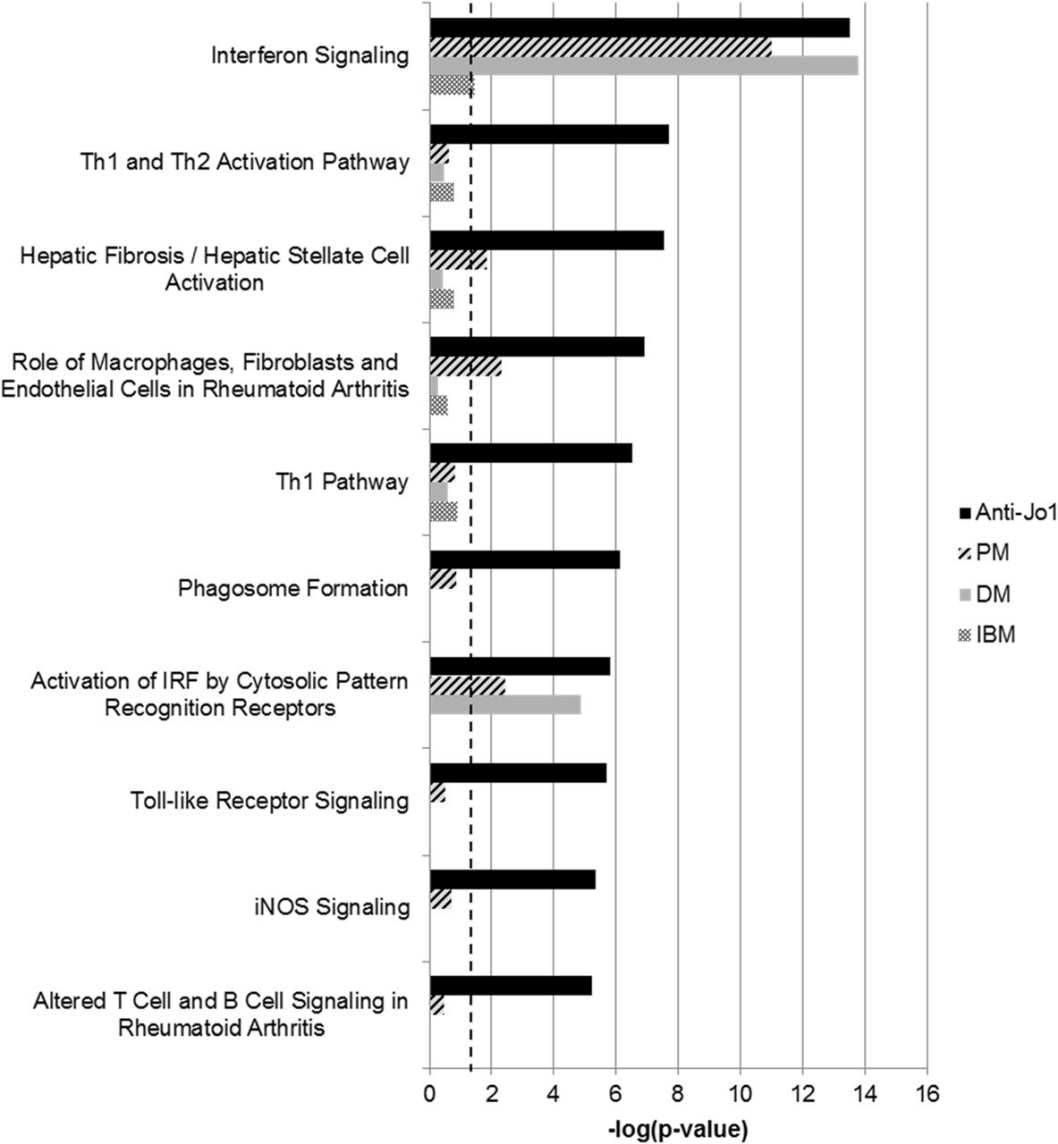
Top ten canonical pathways enriched for dysregulation in anti-Jo1 positive samples compared to controls *PM* Polymyositis, *DM* Dermatomyositis, *IBM* Inclusion Body Myositis, *Anti-Jo1* Anti-Jo1 autoantibody positive subset of PM and DM, *Th* T-helper cell, *IRF* Interferon regulatory factor, *NF-κB*, nuclear factor kappa-light-chain-enhancer of activated B cells, *iNOS* Inducible nitric oxide synthase. Data generated using QIAGEN’s Ingenuity Pathway Analysis ‘Canonical Pathways’ tool on differential expression data from RNA sequencing of whole blood from 7 PM, 7 DM, 5 IBM and 5 non-myositis controls. The dashed line indicates the significance threshold of –log *p*-value > 1.3 (*p*-value < 0.05) for over-representation of dysregulated genes in the pathway

Similarly, when IPA was used to identify canonical pathways with an over-representation of DE genes ‘Interferon signalling’ was the most significantly dysregulated canonical pathway for the PM, DM and anti-Jo1 subgroups and predicted to be upregulated in all three groups (−log *p-*value> 1.3 significant for pathway dysregulation and a z-score > 2 or < − 2 significant for predicted activation or inhibition, respectively) (Fig. 2, Additional file 5). The most significant pathways for PM were interferon or interleukin (IL-6, IL-15 and IL-7) related and highlighted roles for JAK kinases in the signalling of both of these groups of cytokines. The five most significant pathways for DM were related to interferon signalling and anti-viral innate immunity; particularly the role of pattern recognition receptors, interferon regulatory factors and STAT3 (Additional file 5). Grouping by anti-Jo1 positivity similarly identified pathways related to interferon, interleukin, JAK kinases and anti-viral innate immunity. Notably, the anti-Jo1 subgroup had a higher ratio of dysregulated molecules to total molecules in the ‘Interferon signalling’ pathway, a higher proportion of molecules exclusive to type II interferon signalling, including upregulation of *Interferon gamma receptor 1*, and a higher activation score (z-score) compared to the other clinical groups. Additionally, the anti-Jo1 subgroup analysis highlighted T-helper cell-related pathways which were not significantly dysregulated in the other subgroups (Fig. 2, Additional file 5).

### RTqPCR highlighted miR-96-5p and targets

The two most significantly DE miRNAs (*p* < 0.001), miR-92a-1-5p and miR-96-5p, and two miRNAs DE in our data (*p* < 0.01) which have previously been reported to be DE in IIM, miR-10a-5p and miR-223-3p [12, 13], were chosen for follow up by RTqPCR in a subset of samples with sufficient remaining RNA (6 PM and 5 DM (including 5 anti-Jo-1 positive), 4 IBM, and 4 controls). MiR-96-5p had the highest degree of differential expression, upregulated for PM, DM and anti-Jo1, consistent with the small RNA sequencing data, although the RTqPCR data was only statistically significant for DM, probably due to small sample size (Additional Table 2). MiR-10a-5p was validated as significantly downregulated in IBM. MiR-92a-1-5p and miR-223-3p were not significantly DE in RTqPCR analysis of these samples.

**Fig. 3:**
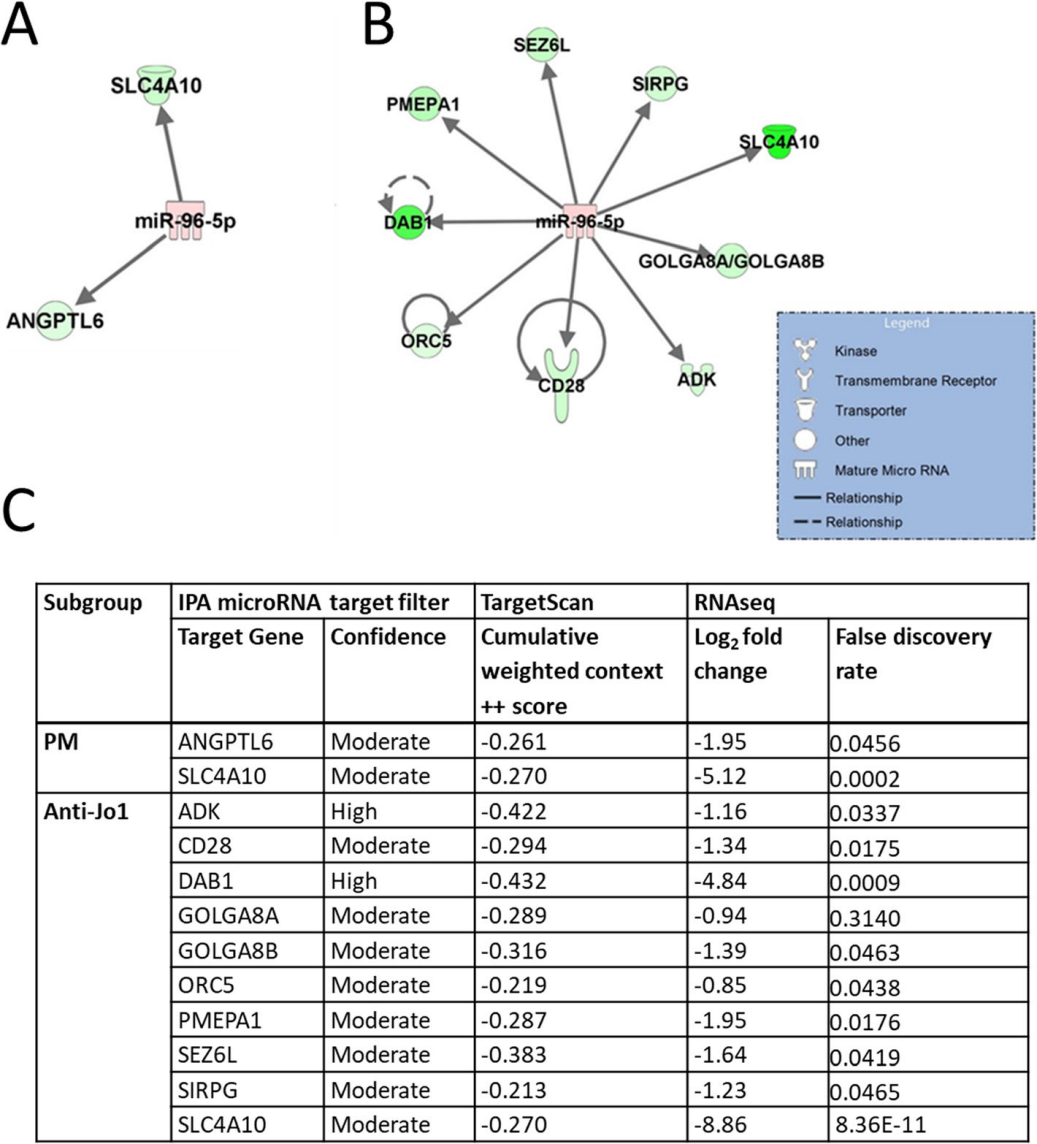
Networks of predicted targets of miR-96-5p downregulated in idiopathic inflammatory myopathy patients compared to controls QIAGEN’s Ingenuity Pathway Analysis (IPA) ‘microRNA target filter’ predicted mRNA targets of miR-96-5p which are downregulated in RNA sequencing of whole blood from (**a**) polymyositis (PM) (*n* = 7) and (**b**) anti-Jo1 positive PM and dermatomyositis (DM) samples (*n* = 5) compared to controls (*n* = 5). No targets were identified in the DM subgroup. Red colouring indicates upregulation, green colouring indicates downregulation and darker shades indicate greater fold change. (**c**) Summary of IPA microRNA target filter predicted targets. Confidence is ‘high’ if the TargetScan database cumulative weighted context ++ score (CWCS) is − 0.4 or below, indicating a predicted repression of at least 25% and ‘moderate’ if the CWCS is − 0.2 to − 0.4, indicating a predicted repression of 13–25%

Predicted targets of miR-96-5p which were downregulated in the RNA sequencing data were identified using the IPA microRNA target filter tool (Fig. 3). A subset of the mRNAs identified with high confidence as predicted targets (*ADK* and *DAB1)* or the most significant dysregulation in the RNA sequencing data *(SLC4A10* and *CD28*) (Fig. 3c) were measured by RTqPCR in samples with remaining RNA (5 PM, 5 DM, (4 anti-Jo1 positive) and 4 controls). *ADK and CD28* were significantly downregulated in anti-Jo1 samples, while *SLC4A10* was significantly downregulated across the PM, DM and anti-Jo-1 subgroups (*p* < 0.05, Table 2). *DAB1* was not expressed at a detectable level.

**Table 2:** RTqPCR of predicted miR-96-5p mRNA targets identified in RNA sequencing in IIM patients versus controls

	Subgroup	RTqPCR			RNAseq	
		Log_**2**_ fold change		T-test	Log_**2**_ fold change	FDR
		Mean	±	***P***-value		
ADK	PM	−0.64	0.87	0.0690	−0.75	0.3520
	DM	−0.49	0.78	0.0960	−0.56	0.8960
	Anti-Jo1	−0.82	0.63	0.0290	−1.16	0.0337
CD28	PM	−1.12	1.59	0.1240	−0.91	0.2780
	DM	−0.63	1.01	0.2840	−0.55	1.0000
	Anti-Jo1	−1.50	1.21	0.0340	−1.34	0.0175
SLC4A10	PM	−4.62	1.89	0.0010	−5.12	0.0002
	DM	−4.02	1.25	0.0010	−2.36	1.0000
	Anti-Jo1	−5.28	0.96	2.40E-05	−8.86	8.36E-11

*PM* Polymyositis, *DM* Dermatomyositis, *Anti-Jo1* Subset of PM and DM with anti-Jo1 autoantibodies

Predicted targets of miR-96-5p which were downregulated in our PM and anti-Jo1 subgroup RNA sequencing data were identified using the IPA microRNA target filter tool and measured by RTqPCR in samples with further remaining RNA (5 PM, 5 DM, (4 anti-Jo1 positive) and 4 controls). Expression fold change was calculated using the 2-∆∆Ct method and then converted to Log_2_ fold change values. *P-*values were calculated using an independent T-test on delta Ct values from each sample (Experimental Ct-Reference Ct) in the subgroups. 

### Transfection of miR-96-5p mimic resulted in downregulation of *ADK*

To investigate the impact of miR-96-5p on the expression of predicted mRNA targets downregulated in IIM in a cell culture model relevant to IIM, human skeletal muscle cells were transfected with miR-96-5p or negative control miRNA mimic. After 24 h, RNA was isolated from cells and expression of miR-96-5p targets *ADK, CD28* and *SLC4A10* was measured using RTqPCR. Expression of *ADK*, but not *CD28* or *SLC4A10*, was significantly downregulated in cells transfected with miR-96-5p (*n* = 5) compared to cells transfected with negative control mimic (*n* = 3) suggesting that *ADK* is suppressed by miR-96-5p in human skeletal muscle (Additional Table 3).

## Discussion

To our knowledge, this is the first study to simultaneously profile both miRNA and mRNA in IIM whole blood using next generation RNA sequencing. Pathway analyses highlighted enrichment of DE genes in innate immune and anti-viral response pathways in PM and DM. Genes in the interferon signalling pathway were upregulated in PM and DM with an increased number of upregulated genes and increased predicted pathway activation in the anti-Jo1 positive subset. This is consistent with previous findings that an interferon score (based on expression of 8 interferon response genes) was higher in whole blood from IIM patients with autoantibodies against RNA-binding proteins, including anti-Jo1, compared to IIM patients with other or no autoantibodies [14]. A larger proportion of the upregulated genes in the anti-Jo1 group were from the type II interferon pathway which is consistent with reports that anti-synthetase syndrome is associated with a prominent type II IFNγ signature in muscle biopsies [15, 16].

The overall number of significant DE genes was much higher in the anti-Jo1 positive subset than in the clinical groups compared to controls. Anti-Jo1 positivity in IIM is associated with a well-defined set of symptoms, termed anti-synthetase syndrome. Therefore, the higher number of DE genes in the anti-Jo1 positive subset may be due in part to a more homogenous sample allowing for more significant differences to be identified when compared to controls. We do not have data on features such as disease activity, disease manifestations and treatments at time of blood draw which may contribute to the striking transcriptomic profile in this subset. T-helper (Th) cell pathways were significantly enriched for dysregulation in the anti-Jo1 subset but not for any of the clinical groups. Since this analysis was performed on whole blood, these findings may reflect greater differences in cell composition in samples from the anti-Jo1 positive group compared to other IIM groups and healthy controls. Analysis of differential gene expression in T-cell subsets from different serological groups within IIM may help to elucidate the role of T-helper cells. RNA sequencing in CD4+ and CD8+ T-cells from PM and DM peripheral blood has shown differential gene expression between PM and DM, especially for CD8+ cells, although the contribution of autoantibody positivity to the differential expression was not investigated [17].

In this study, whole blood was chosen as an abundant source of RNA which may reflect systemic changes in circulating immune cells in IIM. Further work is required to assess how these changes affect the main target tissue of IIM, skeletal muscle, and whether changes in circulating microRNA in particular reflect changes in expression in muscle tissue.

MicroRNA profiling identified 4, 4, 7 and 3 DE miRNAs for PM, DM, IBM and the anti-Jo1 positive subset, respectively, with miR-96-5p significantly DE in PM, DM and anti-Jo1 patients. To our knowledge this is the first miRNA profile in IIM whole blood and therefore it is not surprising that we found significantly differentially expressed microRNA that had not been previously identified in IIM in other tissues. Studies have identified serum miR-7 downregulation as a possible biomarker of PM/DM which is further reduced in patients with ILD [18, 19]. This miRNA may not have been identified in our samples due to differential expression between cell types within whole blood. For example, a study in Sjögren’s syndrome found that miR-146a-5p was upregulated in T-cells but downregulated in B-cells [20] and a study in systemic sclerosis found that miR-618 was upregulated in plasmacytoid dendritic cells but not significantly DE in monocytes [21]. Therefore, to establish which cell type within whole blood is driving these expression changes and whether they reflect changes in expression in skeletal muscle, expression of the identified DE miRNA and mRNA should be measured in different sample types such as plasma, serum and sorted peripheral blood mononuclear cells.

RTqPCR using samples with remaining RNA for four miRNAs found differential expression consistent with the RNA sequencing results for miR-96-5p and miR-10a-5p, although upregulation of miR-96-5p only reached statistical significance for DM, possibly due to small sample sizes. Since miR-96-5p had the largest fold changes in the RTqPCR analyses and has not been identified in IIM before, we investigated the predicted targets of miR-96-5p which were downregulated in the RNA sequencing data. *ADK, SLC4A10* and *CD28* were validated as downregulated in the anti-Jo1 subgroup by RTqPCR. Transfection of human skeletal muscle cells with a miR-96-5p mimic resulted in downregulation of *ADK* but not *SLC4A10* or *CD28* expression, suggesting that miR-96-5p binds and induces degradation of *ADK* in human skeletal muscle. However, binding should be confirmed experimentally and expression analysed in a larger number of whole blood samples and in other tissue types including myositis muscle and sorted blood cells.

miR-96-5p has mainly been studied as an oncogenic miRNA dysregulated in a variety of cancer types including gastric, breast, ovarian and colorectal cancer [22–25]. Adenosine Kinase (ADK) is an enzyme that converts adenosine to adenosine monophosphate and adenosine diphosphate. Both upregulation of miR-96-5p and deficiency of *ADK* have previously been suggested to be associated with mitochondrial dysfunction [26, 27]. Modified reactive oxygen species production as a result of mitochondrial dysfunction may be associated with weakness in IIM [28]. Therefore, if miR-96-5p targeting of ADK is confirmed, this may be an interesting avenue for research in IIM.

The main limitation of this study was the limited sample size due to conservative use of samples from this rare disease. Other limitations include the use of whole blood with variable cellular composition, the higher proportion of males in the control group and the use of samples from patients who have varying degrees of disease severity and were on various treatments for IIM due to the delay to IIM diagnosis, all of which may alter mRNA and miRNA expression.

## Conclusions

Overall, in this study pathway analyses have highlighted enrichment of DE genes in innate immune and anti-viral response pathways in PM and DM and replicated previous findings that interferon signalling is upregulated in PM and DM and more highly upregulated and shifted towards type II interferon signalling in the anti-Jo1 subgroup [14, 15]. In addition, this study highlighted enrichment of dysregulated genes in the T-helper cell pathways in the anti-Jo1 positive subset and indicated a possible role for miR-96-5p regulation of *ADK* in pathogenesis of IIM.

## Supplementary information

**Additional file 1.** Supplementary Methods and Results

**Additional file 2 **Significant RNA sequencing differential expression in polymyositis, dermatomyositis and inclusion body myositis patients versus controls *PM* Polymyositis, *DM* Dermatomyositis, *IBM* Inclusion body myositis, *Anti-Jo1* Subset of PM and DM with anti-Jo1 autoantibodies Data generated by DESeq2 analysis of RNA sequencing data from 7 PM, 7 DM, 5 IBM compared to 5 control whole blood samples. The table columns are gene ids, log_2_ fold change, standard error for log_2_ fold change (lfcSE), statistic value for the null hypothesis (stat), *p*-value, false discovery rate (padj), and gene name.

**Additional file 3 **Significant microRNA sequencing differential expression in polymyositis, dermatomyositis and inclusion body myositis patients versus controls *PM* Polymyositis, *DM* Dermatomyositis, *IBM* Inclusion body myositis, *Anti-Jo1* Subset of PM and DM with anti-Jo1 autoantibodies Data generated by DESeq2 analysis of small RNA sequencing data from 7 PM, 7 DM, 5 IBM compared to 5 control whole blood samples. The table columns are mature microRNA ids, log_2_ fold change, standard error for log_2_ fold change (lfcSE), statistic value for the null hypothesis (stat), *p*-value, false discovery rate (padj), and mature microRNA name.

**Additional file 4 **GOseq analysis of differentially expressed genes in idiopathic inflammatory myopathy patients versus controls *PM* Polymyositis, *DM* Dermatomyositis, *IBM* Inclusion body myositis, *Anti-Jo1* Subset of PM and DM with anti-Jo1 autoantibodies GOseq analysis was performed on significantly differentially expressed genes (FDR < 0.05) from RNA sequencing of 7 PM, 7 DM, 5 IBM and 5 control whole blood samples. The anti-Jo1 subgroup consists of 4 PM and 1 DM sample positive for anti-Jo1 autoantibodies. Columns are gene ontology category, *p*-value for over-representation, Benjamini Hochberg adjusted *p*-value for over-representation (FDR < 0.05 considered significant), *p*-value for under-representation, number of differentially expressed genes in that category, total number of genes in the gene ontology category, description of gene ontology category (term) and type of ontology (BP biological process, MF molecular function, CC cellular component).

**Additional file 5 **Canonical Pathways with an enrichment of significantly differentially expressed genes in idiopathic inflammatory myopathy patients *PM* Polymyositis, *DM* Dermatomyositis, *IBM* Inclusion body myositis, *Anti-Jo1* Subset of PM and DM with anti-Jo1 autoantibodies QIAGEN’s Ingenuity Pathway Analysis (IPA) ‘Canonical Pathways’ analysis was performed on differential expression results from RNA sequencing of 7 PM and 7 DM (5 anti-Jo1 autoantibody positive), 5 IBM and 5 control whole blood samples. Columns are IPA defined canonical pathway, −log(*p*-value) for over-representation of dysregulated molecules in the pathway (> 1.3 is considered significant), ratio of dysregulated molecules to total molecules in pathway, activation score (z-score > 2 is significant predicted activation, <− 2 is significant predicted inhibition, not determined (ND) when IPA algorithm unable to generate a score) and a list of dysregulated molecules in the pathway.

**Additional file 6: Table S1.** Assays used in RTqPCR experiments The assay IDs for the TaqMan® Advanced miRNA assays and gene expression assays used to assess reference genes (‘Reference selection’), for validation of microRNA (‘miRNA validation’) and targets (‘mRNA validation’) found to be dysregulated in RNA sequencing of whole blood from idiopathic inflammatory myopathy patients compared to controls and used to assess expression in human skeletal muscle cells transfected with miRNA mimic compared to cells transfected with negative control miRNA mimic (‘Transfection’). **Table S2.** RTqPCR to validate microRNA identified in RNA sequencing in idiopathic inflammatory myopathy patients versus controls *PM* Polymyositis, *DM* Dermatomyositis, *IBM* Inclusion body myositis, *Anti-Jo1* Subset of PM and DM with anti-Jo1 autoantibodies, *IIM* Idiopathic inflammatory myopathy, *DE* Differentially expressed RTqPCR was performed on total RNA from 6 PM and 5 DM (including 5 anti-Jo-1), 4 IBM, and 4 control whole blood samples. The RTqPCR results are presented next to the RNA sequencing results for these microRNA for comparison. Expression fold change was calculated using the 2^-∆∆Ct^ method and then converted to Log_2_ fold change values. *P*-values were calculated using an independent T-test on delta Ct values from each sample (Experimental Ct-Reference Ct) in the subgroups. **Table S3.** RTqPCR results for skeletal muscle cells transfected with miR-96-5p mimic compared to controls RTqPCR assays for the expression of predicted miR-96-5p mRNA targets were performed on total RNA extracted from human skeletal muscle cells transfected with miR-96-5p miRNA mimic (*n* = 5) and cells transfected with negative control miRNA mimic (*n* = 3). Expression fold change was calculated using the 2^-∆∆Ct^ method and then converted to Log_2_ fold change values. *P-*values were calculated using an independent T-test on delta Ct values from each sample (Experimental Ct-Reference Ct).

## Data Availability

The datasets generated and analysed during the current study are available in NCBI’s Gene Expression Omnibus (Edgar et al., 2002) and are accessible through GEO Series accession number GSE125977 (http://www.ncbi.nlm.nih.gov/geo/query/acc.cgi?acc=GSE125977).

## Abbreviations

IIM: Idiopathic inflammatory myopathy
ADK: Adenosine kinase
cDNA: Complementary DNA
DAB1: DAB adaptor protein 1
DE: Differentially expressed
DM: Dermatomyositis
FDR: False discovery rate
IBM: Inclusion body myositis
IPA: Ingenuity pathway analysis
miRNA: MicroRNA
MRTB: Myositis research tissue bank
PM: Polymyositis
PRDM4: PR Domain Zinc Finger Protein 4
RTqPCR: Reverse transcription quantitative polymerase chain reaction
SLC4A10: Solute carrier family 4 member 10
UBE2D2: Ubiquitin conjugating enzyme E2 D2
