# Supplementary material for: MicroRNA and mRNA profiling in the idiopathic inflammatory myopathies
Source: BMC Rheumatol. 2020 Jun 10;4:25. doi: 10.1186/s41927-020-00125-8 (PMC7285612; doi:10.1186/s41927-020-00125-8)
Supplement: Supplementary file 1 — Additional file 1. Supplementary Methods and Results [file 41927_2020_125_MOESM1_ESM.docx]

# Supplementary methods and results

## RNA sequencing analysis quality control

### FastQC Quality Control

FastQC was used to produce quality reports for each sample (read1 and read 2). All samples passed the quality control checks with per base sequence quality score >25, per sequence quality score (Phred score)>27, per base N content <5% and adapter content <5%.

### Principle components analysis

Unsupervised clustering in principle components analysis did not cluster the samples by subgroups indicating that the non-myositis controls are not significantly different to IIM subgroups (Supplementary Figure 1). There was no obvious clustering to indicate batch effects from RNA isolation, cDNA library preparation or pooling in sequencing lanes.


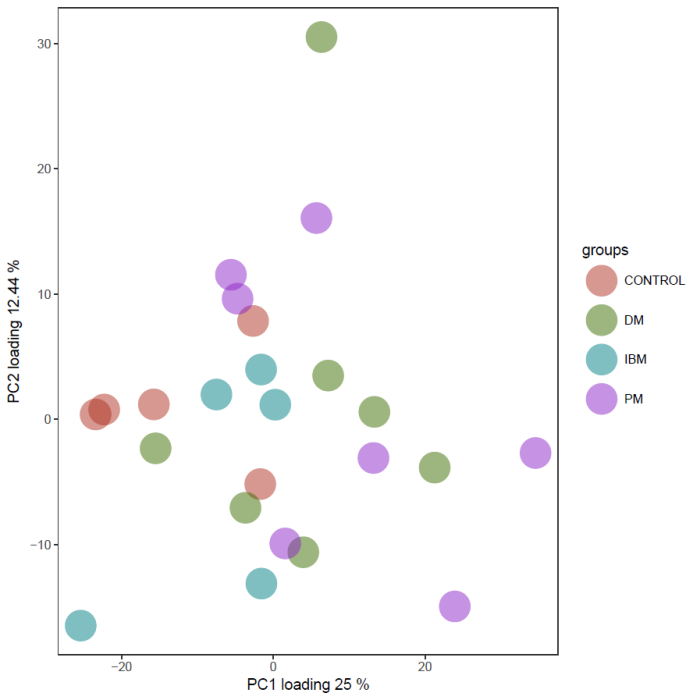

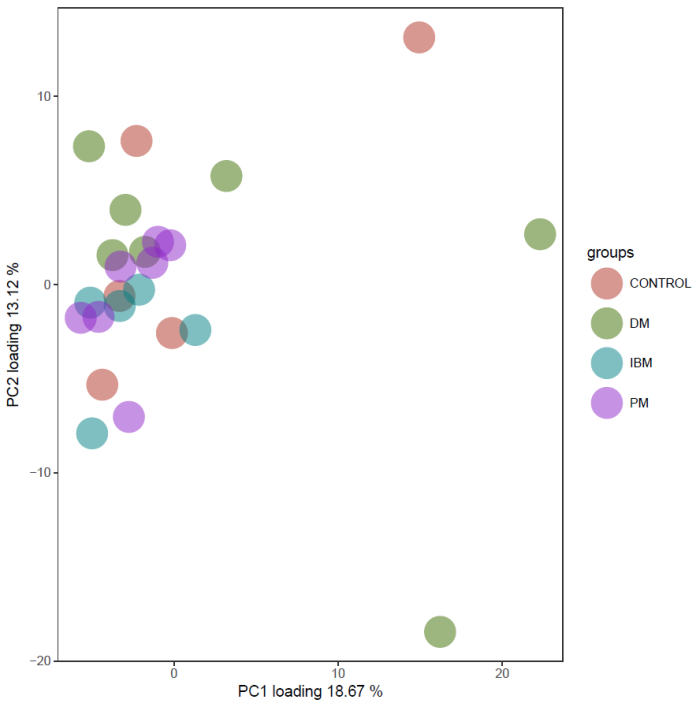


A B

**Supplementary Figure 1 Principle component analysis for A) mRNA and B) miRNA sequencing**

*DM* dermatomyositis, *IBM* inclusion body myositis, *PM* polymyositis, *PC* principle component

## Gene ontology and pathway analyses

The R package ‘GOseq’ was used to analyse lists of significantly DE genes and identify over or under-represented gene ontology terms. GOseq corrects for the bias towards longer transcripts in RNA sequencing data. For this analysis median mature mRNA transcript lengths for each gene from UCSC hg38 were used. Benjamini Hochberg adjusted p-value<0.05 for under or over-represented terms was considered significant.

QIAGEN’s Ingenuity Pathway Analysis (IPA) (IPA®, QIAGEN Redwood City, [www.qiagen.com/ingenuity](http://www.qiagen.com/ingenuity)) ‘Canonical Pathways’ tool was used to identify pathways with an enrichment of significantly DE genes. The analysis uses Fisher’s exact test right-tailed to assign significance and one or more key molecules and their causal relationships are used in an algorithm to calculate an activation score (z-score) for the pathway. A –log p-value >1.3 for enrichment of DE genes in a pathway, z-score >2 for activation or <-2 for inhibition is considered significant.

The IPA ‘MicroRNA Target Filter’ tool was used to match DE miRNA to predicted or experimentally observed mRNA targets with opposite expression changes in the mRNA RNA sequencing data. Predicted mRNA targets are from the TargetScan database and assigned a confidence of ‘high’ if the cumulative weighted context ++ score (CWCS) is -0.4 or below, indicating a predicted repression of at least 25% and ‘moderate’ if the CWCS is -0.2 to -0.4, indicating a predicted repression of 13-25%. The CWCS is a prediction score based on 14 features of the microRNA, the seed sequence and the target mRNA [12]. ‘Experimentally observed’ targeting is from the TarBase and miRecords databases.

## Selection of reference genes for RTqPCR

Reference microRNAs and mRNAs were used for normalisation of RTqPCR results. Two microRNAs (miR-503-5p and miR-425-5p) were selected based on RNAseq results with an average p-value>0.5 and average log_2_ fold change <0.25 in DE analysis for PM, DM and IBM subgroups compared to controls.

For mRNAs five reference gene candidates (*ERCC6, UBE4A, RNF20, UBE2D2* and *PRDM4*) were tested using TaqMan Gene expression assays to perform RTqPCR on all samples used in the mRNA RTqPCR (*n*=16). The suitability of these reference microRNAs was assessed using GeNorm, a Visual Basic Application for Microsoft Excel which uses an algorithm to determine the most stably expressed genes across a group of samples. The gene-stability measure, *M*, is the average pairwise variation of that gene compared to the other reference genes.The two reference genes with the lowest M scores (*UBE2D2* and *PRDM4*) were selected*.* *UBE2D2* and *PRDM4* remained the two lowest scoring genes upon stepwise exclusion of the highest scoring gene in each group.

## Cell line

An immortalised human skeletal muscle cell line, generated from primary human myoblasts (biopsy sample of semitendinosus muscle from a 25 year old male donor), was cultured in standard conditions (5% CO_2_, 37 °C) in growth medium (Medium-199 (Sigma-Aldrich): Dulbecco’s modified eagles medium (Lonza) supplemented with 20% foetal bovine serum (Gibco), 1% penicillin-streptomycin, 1% L-glutamine (Lonza), 10 μg/mL gentamicin, 25 ng/mL fetuin (Sigma-Aldrich), 0.5 ng/mL human fibroblast growth factor, 5 ng/mL human epidermal growth factor, 2.5 ng/mL human hepatocyte growth factor (Gibco), 0.2 μg/mL dexamethasone (Sigma-Aldrich), 5 μg/mL insulin). Cells were incubated until 80% confluence and sub-cultured using Trypsin/EDTA.
