# Supplementary material for: MicroRNA and mRNA profiling in the idiopathic inflammatory myopathies
Source: BMC Rheumatol. 2020 Jun 10;4:25. doi: 10.1186/s41927-020-00125-8 (PMC7285612; doi:10.1186/s41927-020-00125-8)
Supplement: Supplementary file 6 — Additional file 6: Table S1. Assays used in RTqPCR experiments The assay IDs for the TaqMan® Advanced miRNA assays and gene expression assays used to assess reference genes (‘Reference selection’), for validation of microRNA (‘miRNA validation’) and targets (‘mRNA validation’) found to be dysregulated in RNA sequencing of whole blood from idiopathic inflammatory myopathy patients compared to controls and used to assess expression in human skeletal muscle cells transfected with miRNA mimic compared to cells transfected with negative control miRNA mimic (‘Transfection’). Table S2. RTqPCR to validate microRNA identified in RNA sequencing in idiopathic inflammatory myopathy patients versus controls PM Polymyositis, DM Dermatomyositis, IBM Inclusion body myositis, Anti-Jo1 Subset of PM and DM with anti-Jo1 autoantibodies, IIM Idiopathic inflammatory myopathy, DE Differentially expressed RTqPCR was performed on total RNA from 6 PM and 5 DM (including 5 anti-Jo-1), 4 IBM, and 4 control whole blood samples. The RTqPCR results are presented next to the RNA sequencing results for these microRNA for comparison. Expression fold change was calculated using the 2-∆∆Ct method and then converted to Log2 fold change values. P-values were calculated using an independent T-test on delta Ct values from each sample (Experimental Ct-Reference Ct) in the subgroups. Table S3. RTqPCR results for skeletal muscle cells transfected with miR-96-5p mimic compared to controls RTqPCR assays for the expression of predicted miR-96-5p mRNA targets were performed on total RNA extracted from human skeletal muscle cells transfected with miR-96-5p miRNA mimic (n = 5) and cells transfected with negative control miRNA mimic (n = 3). Expression fold change was calculated using the 2-∆∆Ct method and then converted to Log2 fold change values. P-values were calculated using an independent T-test on delta Ct values from each sample (Experimental Ct-Reference Ct). [file 41927_2020_125_MOESM6_ESM.docx]

**Additional Table 1. Assays used in RTqPCR experiments**

| Gene/MicroRNA name | Assay ID | Experiment(s) |
| --- | --- | --- |
| hsa-miR-96-5p | 478215_mir | miRNA validation  Transfection |
| hsa-miR-223-3p | 477983_mir | miRNA validation |
| hsa-miR-370-3p | 478326_mir | miRNA validation |
| hsa-miR-92a-1-5p | 479205_mir | miRNA validation |
| hsa-miR-146a-5p | 478399_mir | miRNA validation |
| hsa-miR-146b-5p | 478513_mir | miRNA validation |
| hsa-miR-10a-5p | 479241_mir | miRNA validation |
| hsa-miR-140-5p | 477909_mir | miRNA validation |
| hsa-miR-30c-1-3p | 479412_mir | miRNA validation |
| hsa-miR-145-5p | 477916_mir | miRNA validation |
| hsa-miR-503-5p | 478143_mir | miRNA validation  Transfection  Reference |
| hsa-miR-425-5p | 478094_mir | miRNA validation  Transfection  Reference |
| *SLC4A10* | Hs01084900_m1 | mRNA validation  Transfection |
| *ADK* | Hs00417073_m1 | mRNA validation  Transfection |
| *CD28* | Hs01007422_m1 | mRNA validation  Transfection |
| *DAB1* | Hs00221518_m1 | mRNA validation |
| *PRDM4* | Hs00183764_m1 | mRNA validation  Transfection  Reference |
| *UBE2D2* | Hs00366152_m1 | mRNA validation  Transfection  Reference |
| *ERCC6* | Hs00972920_m1 | Reference selection |
| *UBE4A* | Hs00191204_m1 | Reference selection |
| *RNF20* | Hs00219623_m1 | Reference selection |

The assay IDs for the TaqMan® Advanced miRNA assays and gene expression assays used to assess reference genes (‘Reference selection’), for validation of microRNA (‘miRNA validation’) and targets (‘mRNA validation’) found to be dysregulated in RNA sequencing of whole blood from idiopathic inflammatory myopathy patients compared to controls and used to assess expression in human skeletal muscle cells transfected with miRNA mimic compared to cells transfected with negative control miRNA mimic (‘Transfection’)

**Additional Table 2. RTqPCR to validate microRNA identified in RNA sequencing in idiopathic inflammatory myopathy patients versus controls**

| MicroRNA | Subgroup | RTqPCR Results | | | RNAseq Results | |
| --- | --- | --- | --- | --- | --- | --- |
|  |  | **Log_2_ fold change** | | **T test** | **Log_2_ fold change** | **P-value** |
|  |  | **Mean** | **±** | **P-value** |  |  |
| hsa-miR-92a-1-5p | PM | 0.32 | 1.73 | 0.6350 | 1.50 | 0.0003 |
|  | DM | -0.11 | 0.76 | 0.8760 | 0.52 | 0.3870 |
|  | IBM | -0.08 | 1.20 | 0.9240 | 1.88 | 0.0004 |
|  | Anti-Jo1 | 0.62 | 1.46 | 0.2960 | 1.48 | 0.0024 |
| hsa-miR-96-5p | PM | 1.69 | 1.56 | 0.0930 | 1.27 | 0.0005 |
|  | DM | 2.15 | 1.23 | 0.0330 | 1.43 | 0.0016 |
|  | IBM | 0.86 | 1.08 | 0.2770 | 0.15 | 0.6860 |
|  | Anti-Jo1 | 1.72 | 1.49 | 0.0900 | 1.40 | 0.0003 |
| hsa-miR-10a-5p | PM | -1.35 | 1.91 | 0.0720 | -1.00 | 0.0320 |
|  | DM | -0.52 | 1.25 | 0.2450 | -0.60 | 0.1500 |
|  | IBM | -1.05 | 0.72 | 0.0010 | -0.99 | 0.0094 |
|  | Anti-Jo1 | -1.23 | 1.32 | 0.0380 | -0.84 | 0.1110 |
| hsa-miR-223-3p | PM | 0.07 | 1.75 | 0.9390 | 0.81 | 0.0115 |
|  | DM | 0.62 | 1.06 | 0.1560 | 0.92 | 0.0197 |
|  | IBM | -0.54 | 0.90 | 0.2580 | 0.51 | 0.0651 |
|  | Anti-Jo1 | 0.26 | 1.72 | 0.7830 | 1.02 | 0.0042 |

*PM* Polymyositis, *DM* Dermatomyositis, *IBM* Inclusion body myositis, *Anti-Jo1* Subset of PM and DM with anti-Jo1 autoantibodies, *IIM* Idiopathic inflammatory myopathy, *DE* Differentially expressed

RTqPCR was performed on total RNA from 6 PM, 5 DM, 4 IBM, 5 anti-Jo1 and 4 control whole blood samples. The RTqPCR results are presented next to the RNA sequencing results for these microRNA and genes for easy comparison. Expression fold change was calculated using the 2^-∆∆Ct^ method and then converted to Log_2_ fold change values. P-values were calculated using an independent T-test on delta Ct values from each sample (Experimental Ct-Reference Ct) in the subgroups.

**Additional Table 3. RTqPCR results for skeletal muscle cells transfected with miR-96-5p mimics compared to controls**

|  | Expression fold change | | | Log_2_ fold change | | T-test |
| --- | --- | --- | --- | --- | --- | --- |
|  | **2^-∆∆Ct^** | **Min** | **Max** | **Mean** | **±** | **p-value** |
| ADK | 0.596 | 0.539 | 0.660 | -0.746 | 0.146 | 0.000424 |
| CD28 | 0.580 | 0.393 | 0.855 | -0.786 | 0.560 | 0.096 |
| SLC4A10 | 1.739 | 0.448 | 6.746 | 0.798 | 1.956 | 0.612 |

RTqPCR assays for the expression of predicted miR-96-5p mRNA targets were performed on total RNA extracted from human skeletal muscle cells transfected with miR-96-5p miRNA mimics (*n*=5) and cells transfected with negative control miRNA mimics (*n*=3). Expression fold change was calculated using the 2^-∆∆Ct^ method and then converted to Log_2_ fold change values. P-values were calculated using an independent T-test on delta Ct values from each sample (Experimental Ct-Reference Ct) in the subgroups.
